# Supplementary figures and images for: Heterologous Production of 1-Tuberculosinyladenosine in Mycobacterium kansasii Models Pathoevolution towards the Transcellular Lifestyle of Mycobacterium tuberculosis
Source: mBio. 2020 Oct 20;11(5):e02645-20. doi: 10.1128/mBio.02645-20 (PMC7587436; doi:10.1128/mBio.02645-20)

7H9 pH 6.6

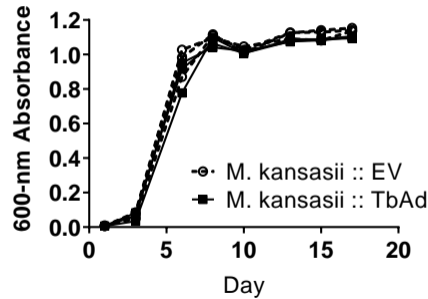

7H9 pH 5.4

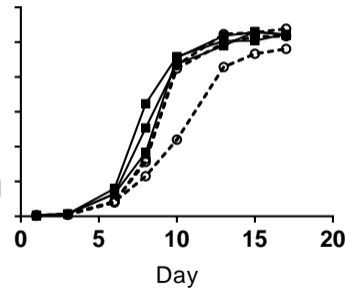

7H9 pH 5.2

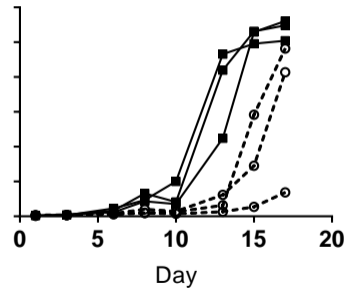

7H9 pH 5.1

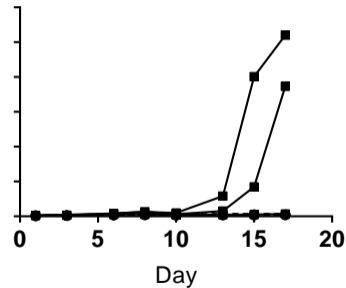

7H9 pH 5.0

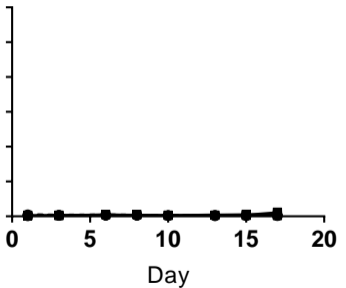

Supplement: FIG S1 [file mBio.02645-20-sf001.pdf]

a

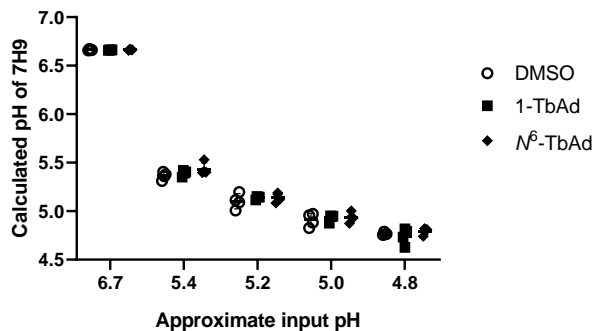

b

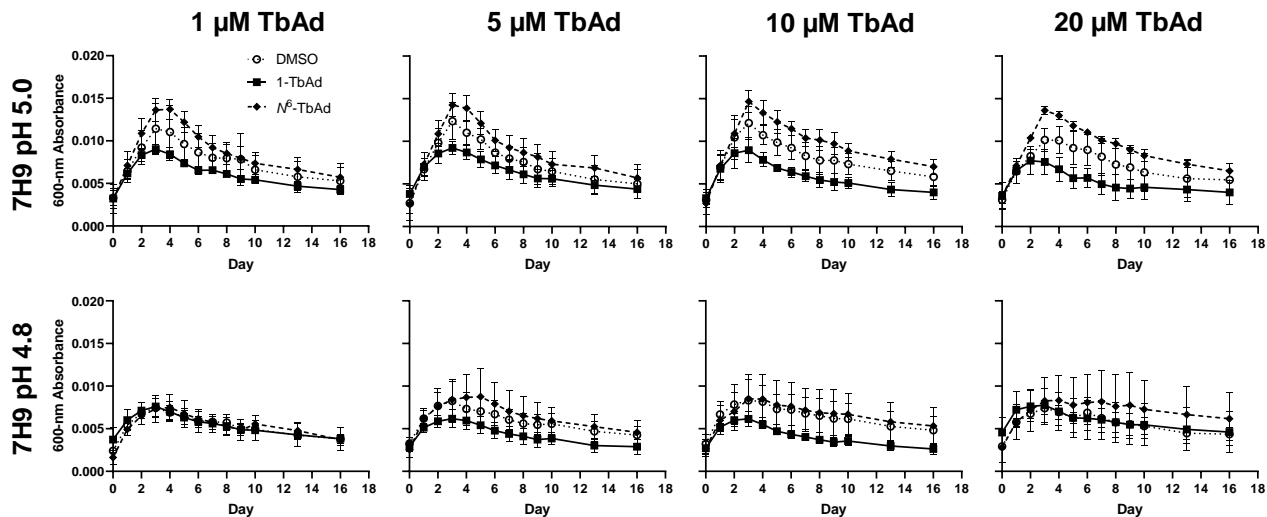

Supplement: FIG S2 [file mBio.02645-20-sf002.pdf]

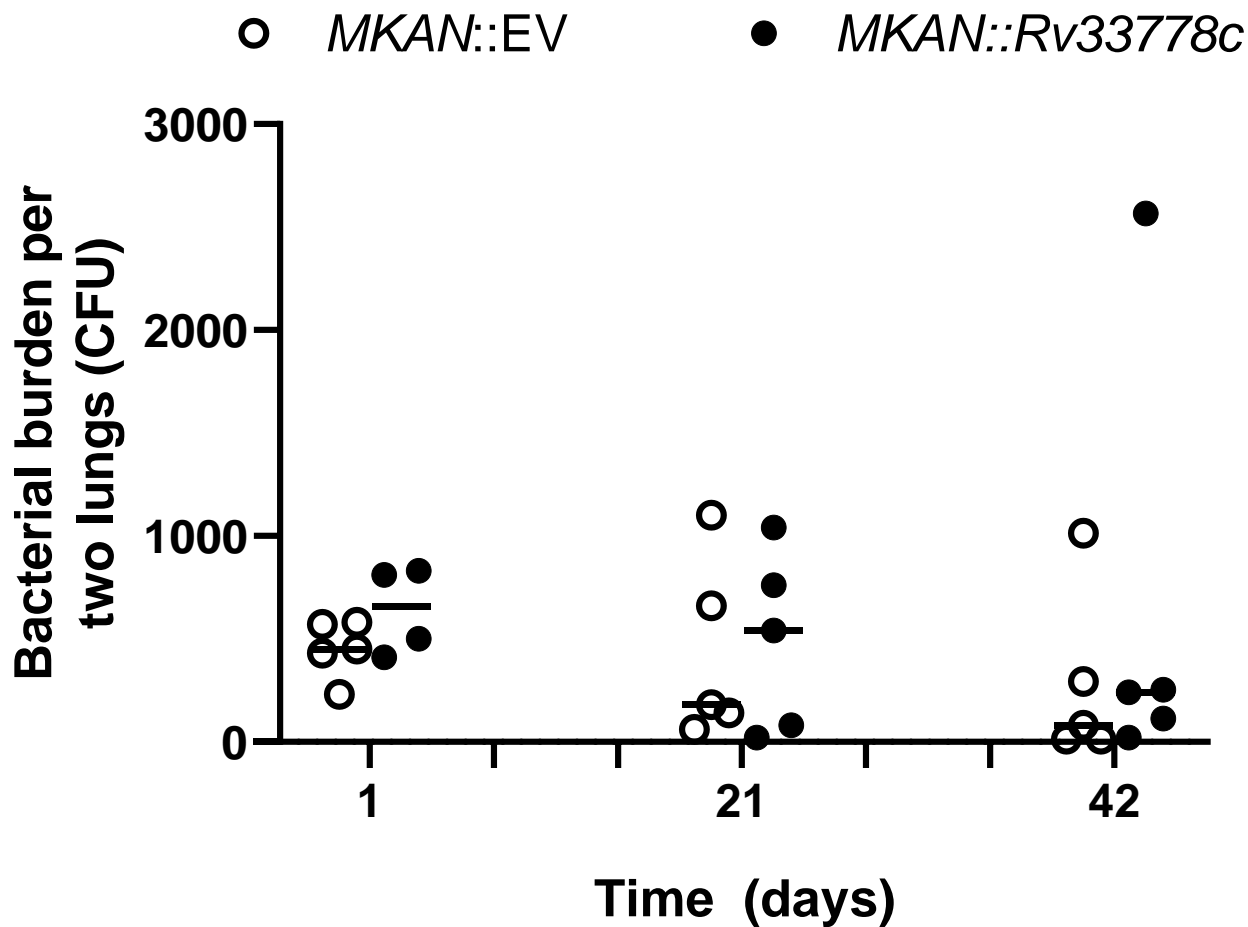

Supplement: FIG S3 [file mBio.02645-20-sf003.pdf]

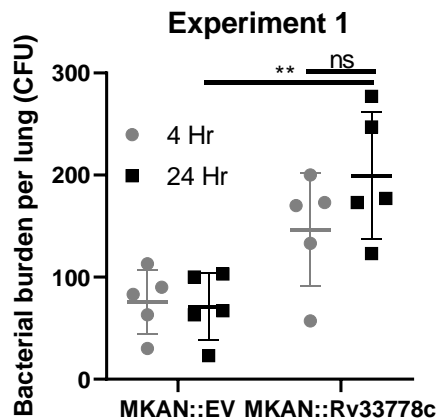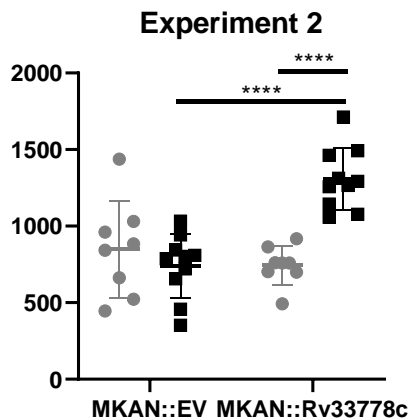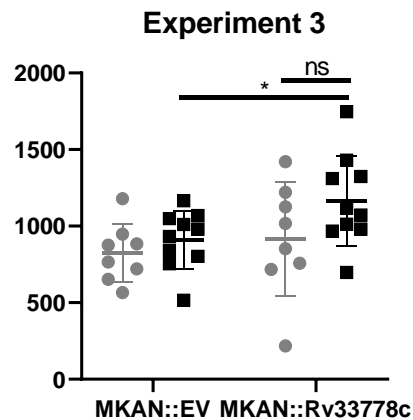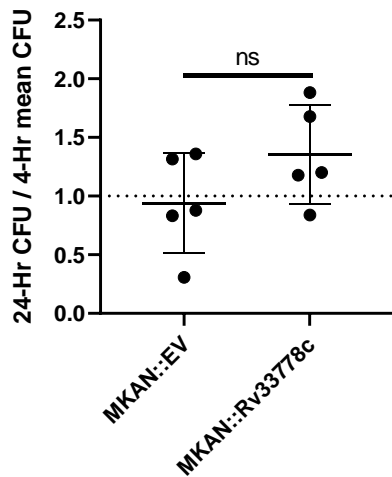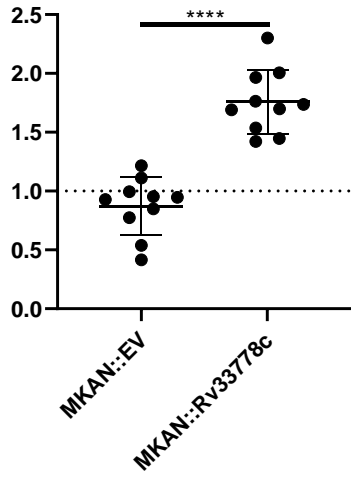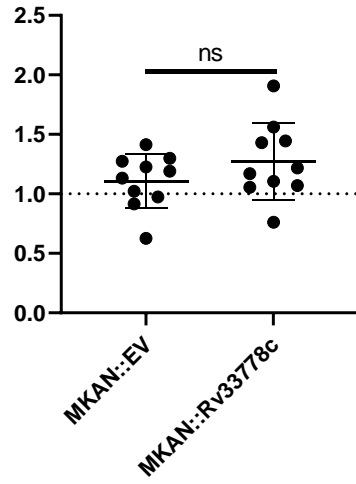

Supplement: FIG S4 [file mBio.02645-20-sf004.pdf]

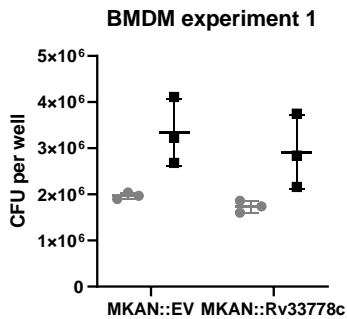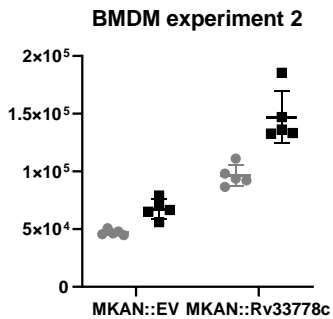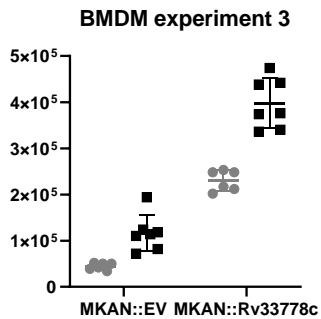

● 4 Hr  
■ 24 Hr

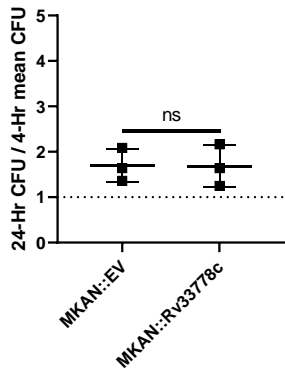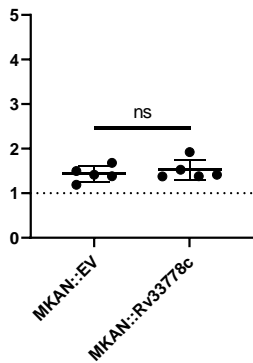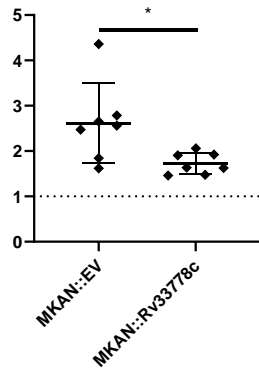

**BMDM experiments 1+2+3**

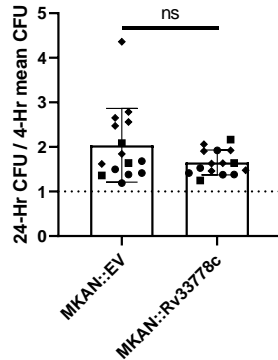

Supplement: FIG S5 [file mBio.02645-20-sf005.pdf]

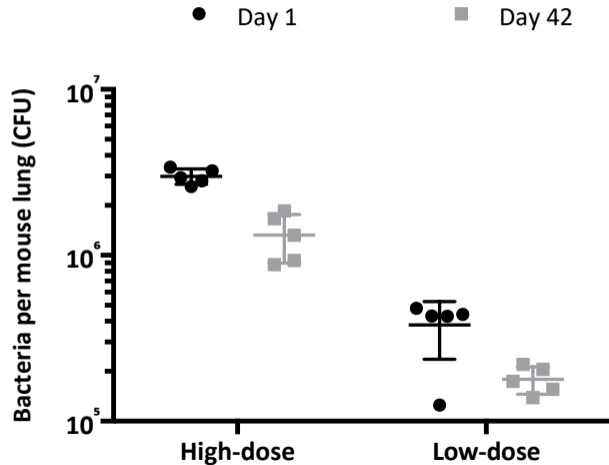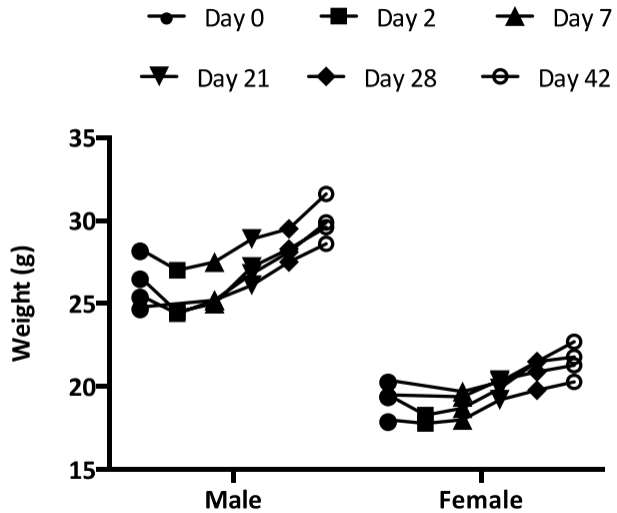

Supplement: FIG S6 [file mBio.02645-20-sf006.pdf]

a

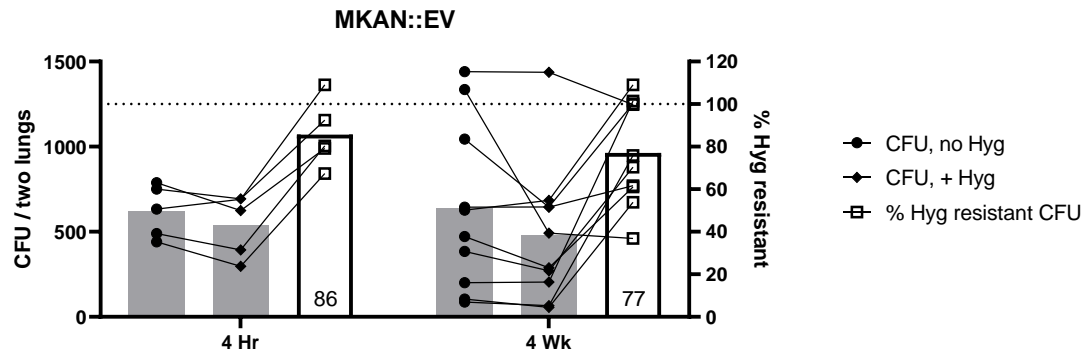

b

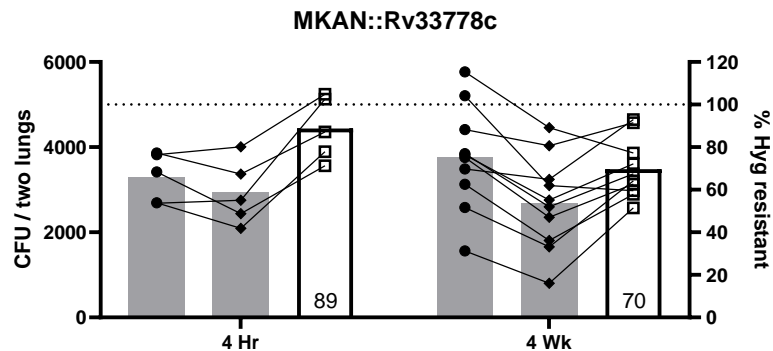

c

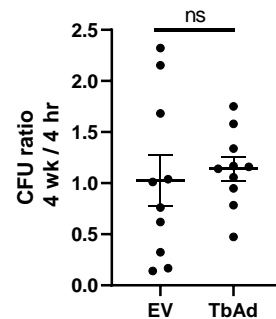

Supplement: FIG S7 [file mBio.02645-20-sf007.pdf]
