# Supplementary material for: Heterologous Production of 1-Tuberculosinyladenosine in Mycobacterium kansasii Models Pathoevolution towards the Transcellular Lifestyle of Mycobacterium tuberculosis
Source: mBio. 2020 Oct 20;11(5):e02645-20. doi: 10.1128/mBio.02645-20 (PMC7587436; doi:10.1128/mBio.02645-20)
Supplement: TABLE S1 [file mBio.02645-20-st001.pdf]

Primer name	Primer sequence	Use
**BamHI-Rv3377-78c-F**	CGGGATCCATGAACTTGGTTAGCGAAAAAG	Cloning
**HindIII-Rv3377-78c-R**	CCAAGCTTTCATTGGTTACTCTCATCGACC	Cloning
**gDNA-Rv3377c-check-F**	TCGAGCACAGCCTATGACAC	Genotyping
**gDNA-Rv3377c-check-R**	ACCGATCCATTTGTCTCCTG	Genotyping
**gDNA-Rv3378c-check-F**	CACGAGGTCCACGTTCTTTT	Genotyping
**gDNA-Rv3378c-check-R**	CAACCCACACCGAAAACTCT	Genotyping
**Rv3377-78cSeq-F1**	GCCTTTGAGTGAGCTGATACC	Sanger sequencing
**Rv3377-78cSeq-F2**	GCTGGTTTCACCTCGAATG	Sanger sequencing
**Rv3377-78cSeq-F3**	GTCATTTCCGGCCCAAAC	Sanger sequencing
**Rv3377-78cSeq-F4**	CTTGGCGTACATCTCATCGA	Sanger sequencing
**Rv3377-78cSeq-R1**	GATAATCTCTCTCCGCGTG	Sanger sequencing
**Rv3377-78cSeq-R2**	CTTATTCGACGTGAGGCTG	Sanger sequencing
**Rv3377-78cSeq-R3**	CATCGTAGGCCACACTTGTG	Sanger sequencing
**Rv3377-78cSeq-R4**	CTGTCGTTCACGGCTCTAG	Sanger sequencing
**qMKANSigA-F**	CGGAGAAGGTGCTCGAAATC	qRT-PCR
**qMKANSigA-R**	TGGTCTGGTCCAGCGAGATC	qRT-PCR
**qRv3377c-F**	CAAGCTCTGGCGCATTGG	qRT-PCR
**qRv3377c-R**	GATCTGCGCCGACAAGGA	qRT-PCR
**qRv3378c-F**	CAACGATGCGGCTGAGTCT	qRT-PCR
**qRv3378c-R**	TTACCATGCGTTTCGTTCCA	qRT-PCR
